# Work-Life Balance as a Mediator Between Work Environment and Psychological Well-Being Among University Lecturers and Administrative Staff

**DOI:** 10.21315/mjms-12-2025-853

**Published:** 2026-04-30

**Authors:** Gilbert Pieter De Vendy Turuk, R Pasifikus Christa Wijaya, Audrey Gracelia Riwu, Nicholas Edwin Handoyo

**Affiliations:** 1 Medical Study Program, Faculty of Medicine and Veterinary Medicine, Universitas Nusa Cendana, Kupang, East Nusa Tenggara, Indonesia; 2 Psychology Department, Faculty of Public Health, Universitas Nusa Cendana, Kupang, East Nusa Tenggara, Indonesia; 3 Department of Biomedicine, Faculty of Medicine and Veterinary Medicine, Universitas Nusa Cendana, Kupang, East Nusa Tenggara, Indonesia; 4 Medical Education Department, Faculty of Medicine and Veterinary Medicine, Universitas Nusa Cendana, Kupang, East Nusa Tenggara, Indonesia

**Keywords:** work environment, psychological well-being, work-life balance, higher education, mediation analysis

## Abstract

**Background:**

This study examined the direct and indirect effects of the work environment on psychological well-being (PWB), with work-life balance (WLB) as a mediating variable among lecturers and administrative staff at Universitas Nusa Cendana, Indonesia.

**Methods:**

A cross-sectional quantitative survey was conducted in June 2025 across several faculties and administrative units using validated Indonesian versions of three instruments: the 32-item Work Environment Services Scale, the 15-item WLB Scale, and the 18-item Ryff PWB Scale. A total of 347 respondents participated, comprising 229 lecturers and 118 administrative staff. Data were analysed using SmartPLS 4.0 and PROCESS Macro Model 4 with 5,000 bootstrap resamples.

**Result:**

A total of 347 respondents were included in the analysis. PWB was significantly impacted directly by the work environment (*β* = 0.20, *P* < 0.001). WLB accounted for 54.4% of the total effect and significantly mediated the association between PWB and the work environment (indirect effect *β* = 0.24, *P* < 0.001). The model explained 25.3% of the variance in WLB and 39.2% of the variance in PWB, suggesting a significant role for WLB in the relationship between workplace conditions and employee well-being.

**Conclusion:**

A supportive work environment enhances PWB directly and indirectly through WLB. Strengthening collegial relationships, equitable workload distribution, and WLB-based policies can effectively promote staff well-being in higher education, particularly in eastern Indonesia.

## Introduction

Globally, approximately 970 million people experience mental disorders, accounting for 12.5% of the total disease burden (1). In Southeast Asia, mental disorders are the second leading cause of years lost due to disability (2). Meanwhile, in Indonesia, the prevalence of mental disorders increased from 6.0% in 2013 to 9.8% in 2018 (3). These patterns are influenced by factors such as unemployment, family conflicts, work environment, and socioeconomic stress (4).

Meanwhile, in the era of globalisation and digital transformation, higher education institutions face rapid changes that demand adaptability from lecturers and administrative staff at the core of university operations. They are tasked with curriculum innovation, research, publication, community service, and administrative responsibilities. However, this often leads to excessive workloads and declining psychological well-being (PWB). PWB is defined as a multidimensional construct encompassing self-acceptance, social relationships, autonomy, environmental mastery, life purpose, and personal growth (5–7).

A supportive work environment, both physical (e.g., facilities, lighting, and noise) and non-physical (e.g., interpersonal relations, leadership, and culture), helps enhance productivity, and reduce stress and burnout (8–11). Likewise, work-life balance (WLB), the ability to harmonise professional and personal roles, has a strong positive association with PWB (12, 13).

Numerous studies have examined these relationships, particularly the direct associations between the work environment and PWB, and WLB and PWB (14–19). However, most studies have focused on dyadic (two-variable) relationships without analysing how the three variables interact within a single integrated model. Few studies simultaneously investigate the work environment, WLB, and PWB, especially by positioning WLB as a mediating variable.

Therefore, empirical studies on the interrelations among these three variables in Indonesian higher education, particularly in eastern regions such as East Nusa Tenggara, are scarce. Addressing this gap, this study examines the work environment’s direct and indirect effects on PWB through WLB among lecturers and administrative staff at Universitas Nusa Cendana. The findings can provide valuable empirical insights for developing flexible work policies, resilience training, and institutional support systems to strengthen organisational health and staff well-being.

## Methods

### Study Design and Sample Size

This study employed a quantitative analytical design with a cross-sectional approach to examine the relationship between work environment and PWB, and WLB’s mediating role among lecturers and administrative staff at Universitas Nusa Cendana, Indonesia. The sample size was calculated using the Isaac–Michael formula, resulting in 304 participants with a 5% error margin. A stratified quota sampling technique ensured proportional representation among the university faculty members.

### Inclusion and Exclusion Criteria

Participants included active lecturers and administrative staff who voluntarily consented to participate. The exclusion criteria were incomplete questionnaires, duplicate responses from the same respondent, responses that could not be analysed due to technical problems during submission, and health professionals working at Universitas Nusa Cendana Hospital or affiliated clinics.

### Measurements

Three main variables were measured using validated Indonesian instruments:

- Work Environment Services Scale (WESS) was developed by Patrick and Kareem (20), comprising 32 items across nine dimensions (ethics, autonomy, stress, practices, managerial support, commitment, role clarity, social responsibility, and coworker coherence). Responses were rated on a 5-point Likert scale (1 = strongly disagree to 5 = strongly agree), producing scores between 32 and 160. Negative items were reverse-scored. The scores were classified as very low (< 25th percentile), low (25th percentile), moderate (median–75th percentile), or high (> 75th percentile).
- WLB Scale was developed by Fisher et al. (21) and validated in Indonesia (22, 23). This 15-item instrument covers four dimensions: Work Interference with Personal Life, Personal Life Interference with Work, Work Enhancement of Personal Life (WEPL), and Personal Life Enhancement of Work (PLEW). Each item is rated on a 5-point Likert scale (1 = never to 5 = very often), with total scores ranging from 15 to 75. The classifications followed the following percentile ranges: low (< 25th percentile), moderate (25th to 75th), and high (> 75th percentile).
- The Ryff Psychological Well-Being Scale (RPWBS-18) was adapted from Ryff and Keyes (24) and validated in Indonesia by Humaidah and Mulyono (25). This 18- item scale measures six dimensions: self-acceptance, positive relations, autonomy, environmental mastery, purpose in life, and personal growth. Responses were rated on a 6-point Likert scale (1 = strongly disagree to 6 = strongly agree), with total scores ranging from 18 to 108. Negative items were reverse-scored. The classifications followed the following percentile-based categories: low (< 25th percentile), moderate (25th to 75th), and high ( 75th percentile).

### Data Collection

Primary data were collected via an online self-administered questionnaire using Google Forms in early June 2025. The survey remained open for three weeks, with two weekly reminders disseminated via faculty leaders, WhatsApp groups, and the university’s official Instagram. All participants provided informed consent prior to participation. Secondary data (faculty statistics and contact information) were obtained from university records.

### Data Analysis

Descriptive statistics were used to summarise categorical (frequency and percentage) and numerical variables (mean and standard deviation). The main study variables, work environment, PWB, and WLB, were treated as numerical variables derived from the composite scores of the Likert-scale questionnaire items. Normality was tested using the Kolmogorov–Smirnov test. Mediation analysis was performed using SmartPLS 4.0 and PROCESS Macro Model 4 (26), with 5,000 bootstrap resamples to estimate indirect effects. The effect size (*f*^2^) thresholds were interpreted as small (0.02), medium (0.15), and large (0.35), and *R*^2^ indicated the explained variance of the dependent variables (27). The mediation effect (*v*) was categorised as small (0.01), medium (0.075), or large (0.175) (28).

## Results

### Participants

In total, 354 individuals initially participated in the survey. After applying the inclusion and exclusion criteria, data from 347 respondents were eligible for analysis. Seven responses were excluded due to incomplete or unfinished questionnaire submissions (Figure 1). Among the remaining participants, 229 were lecturers, and 118 were administrative staff members. The respondents’ ages ranged from 24 to 65 years, with a mean age of 39.70 years.

According to the World Health Organization’s age classification, the majority of respondents were adults (25 to 59 years), comprising 327 participants (94.2%), followed by older adults (60 to 99 years) at 19 participants (5.5%) and with only one young adult (20 to 24 years; 0.3%). The sample represents a diverse range of ages, faculties, and employment categories. Table 1 presents the respondents’ demographic characteristics.

### Descriptive Analysis of Study Variables

PWB and WLB scores were categorised into three levels (low, moderate, and high) based on the sample’s percentile cut-off values. Meanwhile, for work environment, the scores were categorised into four groups based on their percentiles: very low, low, medium, and high. The calculated cut-off value for the sample is presented in Table 2. Reliability testing using Cronbach’s alpha indicated high internal consistency: work environment (α = 0.90), WLB (α = 0.87), and PWB (α = 0.75). These categorisations followed measurement recommendations (20, 21, 24).

The majority of respondents perceived their work environment to be in the low category (26.8%) (Figure 2). Overall, the mean scores indicated that both lecturers and administrative staff generally rated their work environment as low. The autonomy dimension had the highest mean (SD) score of 18.10 (3.70), while peer cohesiveness had the lowest mean (SD) score of 7.60 (1.70) (Table 3).

Meanwhile, in terms of WLB, most respondents (51.6%) were in the moderate category (Figure 2). The highest mean (SD) score was observed for the PLEW dimension at 12.30 (2.40), whereas the lowest mean (SD) score was for the WEPL dimension at 7.30 (1.90) (Table 4).

Regarding PWB, most respondents (51.9%) belonged to the moderate category (Figure 2). The self-acceptance dimension had the highest mean (SD) score of 15.60 (2.30), while the autonomy dimension had the lowest mean (SD) score of 13.70 (2.40) (Table 5).

When viewed based on employment status, most lecturers (28.3%) rated their work environment as high. Conversely, among administrative staff, most respondents (38.9%) rated their work environment as low (Figure 2). Both lecturers and administrative staff reported the highest mean (SD) scores in the autonomy dimension and the lowest in the peer cohesiveness dimension (Table 3).

For WLB, most lecturers (47.6%) were in the moderate category. A similar pattern was observed among the administrative staff, with 59.3% in the moderate category (Figure 2). Both groups demonstrated the highest mean score in the PLEW dimension and the lowest in the WEPL dimension (Table 4).

In terms of PWB, nearly half of the lecturers (49.3%) and the majority of the administrative staff (56.7%) were in the moderate category (Figure 2). Both had the same highest scores in the personal growth dimension. However, lecturers scored lowest on purpose in the life dimension, while administrative staff scored lowest on the autonomy dimension (Table 5).

### Mediation Analysis

Mediation analysis was conducted using the PROCESS macro model 4 to examine WLB’s role as a mediator in the relationship between work environment and PWB. A bootstrapping procedure with 5,000 resamples and bias-corrected confidence intervals (CI) was used to estimate the effects. The results showed that the work environment had a significant direct effect on PWB (*β* = 0.20; *P* < 0.001; 95% CI: 0.09, 0.32) (Figure 3). In addition, the work environment had a significant indirect effect on PWB through WLB (*β* = 0.24; *P* < 0.001; 95% CI: 0.19, 0.31). Thus, WLB partially mediated the relationship between work environment and PWB at 54.4% of the total effect, suggesting that more than half of the effect of the work environment on PWB is explained by WLB.

This model explained 39.2% and 25.3% of the variance in PWB and WLB, respectively. The effect size (*f*^2^) for the direct effect of the work environment on PWB was small (*f*^2^ = 0.05), whereas the effects of work environment on WLB (*f*^2^ = 0.33) and WLB on PWB (*f*^2^ = 0.30) were large. The mediation effect size of WLB on the relationship between work environment and PWB was small to moderate (*v* = 0.06). The results are summarised in Table 6.

## Discussion

Focusing on lecturers and administrative staff at Universitas Nusa Cendana, this study examined the work environment’s direct effect on PWB and its indirect effect through WLB as a mediator. It was motivated by the increasing academic and administrative demands in the digital era, which may adversely affect staff well-being, particularly in higher education institutions in eastern Indonesia.

The results showed that the work environment influenced PWB directly (*β* = 0.20, *P* < 0.001) and indirectly through the partial mediation of WLB (*β* = 0.24, *P* < 0.001). Notably, the latter accounted for 54.4% of the total effect. The model explained 39.2% of the variance in PWB and 25.3% in WLB. These findings emphasise that a supportive work environment that promotes WLB plays a crucial role in enhancing PWB within higher education contexts.

### Overview of PWB, WLB, and Work Environment

Notably, 28.3% lecturers rated the work environment to be high, with autonomy scoring the highest. This indicates that many felt empowered to make decisions independently. However, peer cohesiveness received the lowest score, reflecting weaker collegial relationships and limited peer support, which may have reduced motivation and engagement (29, 30).

Meanwhile, most administrative staff members (62.7%) rated their work environment as low or very low. Although they reported high autonomy, the low peer cohesiveness dimension indicates limited peer support and teamwork, which could diminish motivation and PWB. These findings highlight a clear gap in the perception of the work environment between lecturers and administrative staff, and emphasise the need to strengthen social relationships in the workplace to foster a more inclusive and supportive environment.

In general, the majority of respondents among both lecturers and administrative staff perceived their balance between work and personal life as moderate. Among the WLB dimensions, the lowest score was for WEPL, indicating that work did not sufficiently improve the quality of personal life. Conversely, PLEW scored higher, suggesting that personal life factors such as family support and emotional stability positively contributed to job satisfaction (21, 31, 32).

Concerning PWB, both lecturers and administrative staff were generally in the moderate category. Among lecturers, personal growth scored the highest, reflecting a positive self-perception as developing individuals. Meanwhile, purpose in life scored the lowest, indicating uncertainty about meaning and direction in their work, factors often linked to reduced motivation and burnout (33). In contrast, among the administrative staff, personal growth scored the highest, suggesting a sense of self-improvement and adaptability. Meanwhile, autonomy scored the lowest, indicating limited independence in making decisions and performing tasks.

Overall, both lecturers and administrative staff demonstrated moderate levels of WLB and PWB, but perceived the work environment differently: Lecturers tended to rate it positively, while administrative staff rated it low due to limited peer support. Personal growth was the highest-rated dimension for both groups, whereas lecturers faced challenges in their purposes in life, and administrative staff faced challenges in autonomy.

Thus, well-being depends not only on personal development but also on meaningful work, supportive social relationships, and a conducive work environment. Therefore, universities should strengthen their collaborations, foster meaningful engagement, and implement policies which holistically support the well-being of all staff members.

### Mediating Role of WLB

The mediation analysis showed that WLB played a substantial role in linking the work environment to PWB. The indirect effect was significant (*β* = 0.24, *P* < 0.001), accounting for 54.4% of the total effect, indicating a moderate-to-large partial mediation (27). Although the direct effect of the work environment on PWB remained significant (*β* = 0.20, *P* < 0.000), its magnitude was relatively small (*f*^2^ = 0.05). Meanwhile, the effect of WLB on PWB was much stronger (*f*^2^ = 0.30), underscoring WLB’s central role in sustaining well-being among university employees (12).

Clearly, external organisational factors primarily influence individuals’ psychological states through the mediating mechanism of WLB. A supportive work environment marked by collegiality, supervisor support, and a positive climate contributes to well-being; however, its impact becomes more meaningful when employees maintain a balance between work and personal life. Thus, WLB acts as a psychological buffer that enables individuals to manage stress, regulate emotions, and maintain adaptive functioning under pressure (34). Without sufficient balance, even favourable work conditions may not translate into lasting psychological benefits.

In the academic context, where professional and personal demands often overlap, WLB transforms a supportive environment into tangible improvements in mental health and job satisfaction (35). Therefore, institutions must go beyond improving workplace quality by implementing holistic strategies, such as flexible work arrangements, family supportive policies, stress-management programmes, and resilience training, to strengthen employees’ ability to harmonise both life domains (36, 37).

Ultimately, employees who experience better WLB tend to be more resilient, engaged, and productive, engendering stronger organisational outcomes (17). Thus, enhancing PWB requires a dual focus: creating a supportive environment and empowering individuals to sustain WLB through structured interventions (38). Hence, WLB serves as a key mechanism in transforming workplace support into enduring PWB and overall life satisfaction.

### Limitations

First, the sample was limited to faculty members and administrative staff at a single Indonesian university, which may restrict the generalizability of our findings. Although the sample represented all university units, its size and scope may not reflect broader higher education populations.

Second, other potential influences, such as socio-economic conditions, family support, and personality traits, were not examined, which may limit our understanding of the factors shaping PWB.

Overall, our findings should be interpreted with caution. Future research should include larger and more diverse samples, apply longitudinal or experimental approaches, and incorporate additional contextual variables to provide a more comprehensive view of PWB in academic settings.

## Conclusion

This study found that lecturers and administrative staff at Universitas Nusa Cendana generally perceived the work environment as less supportive, with administrative staff rating it lower than lecturers. Still, both groups reported moderate levels of WLB and PWB. The findings indicate that the work environment has both direct and indirect effects on PWB through WLB as a mediating variable. Thus, strengthening collegial support and ensuring a fair workload distribution may help improve both WLB and PWB. Therefore, universities should implement targeted interventions that support WLB, such as flexible work arrangements, counselling services, stress-management programmes, and peer collaboration initiatives, to foster a more supportive and psychologically healthy academic environment.

## Figures and Tables

**Figure 1:**
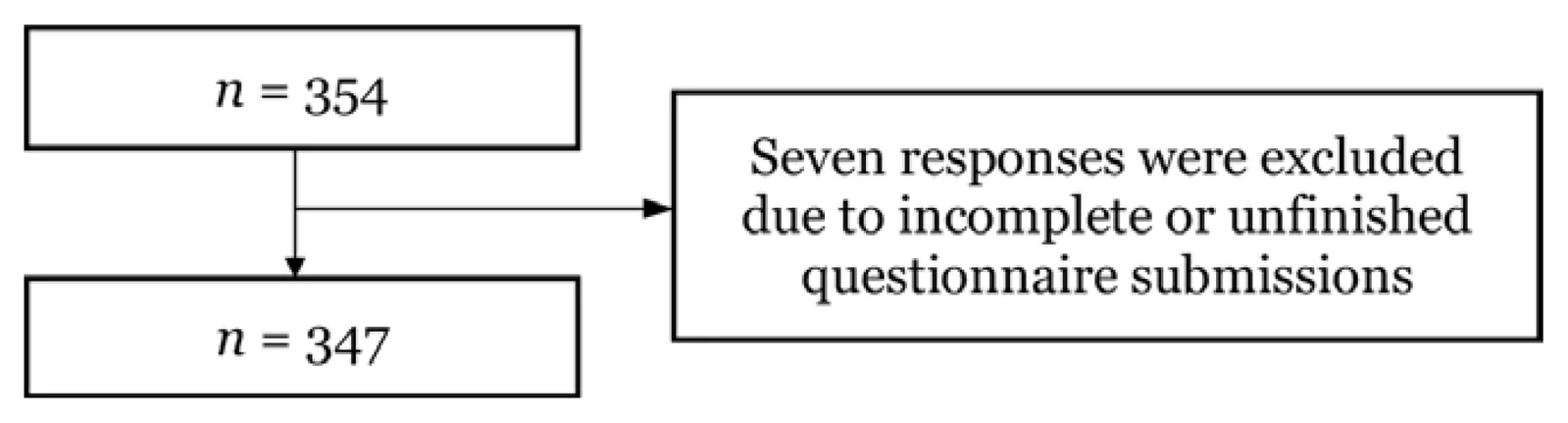
Respondents’ selection process

**Figure 2:**
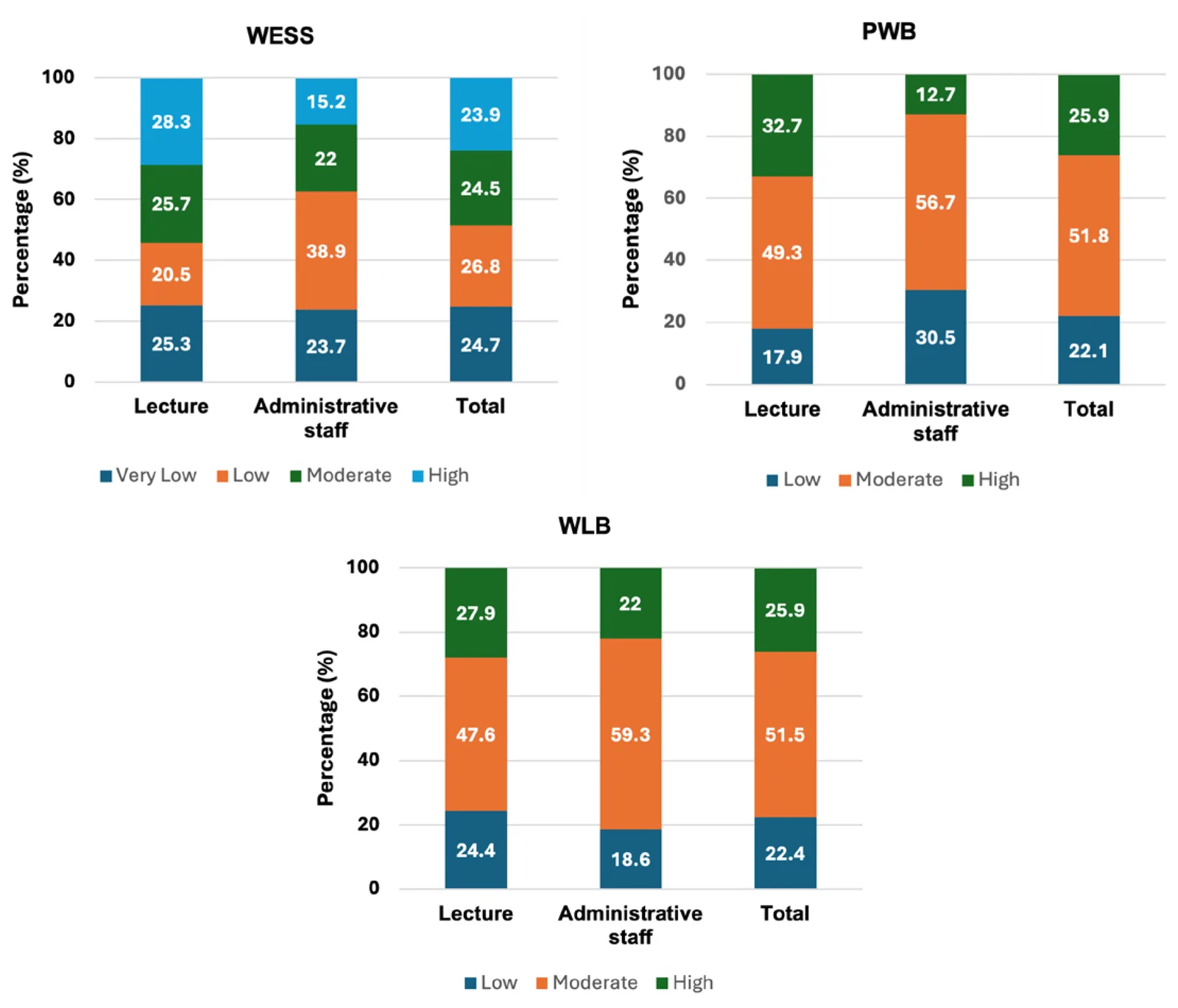
Descriptive analysis of WESS, WLB, and PWB

**Figure 3:**
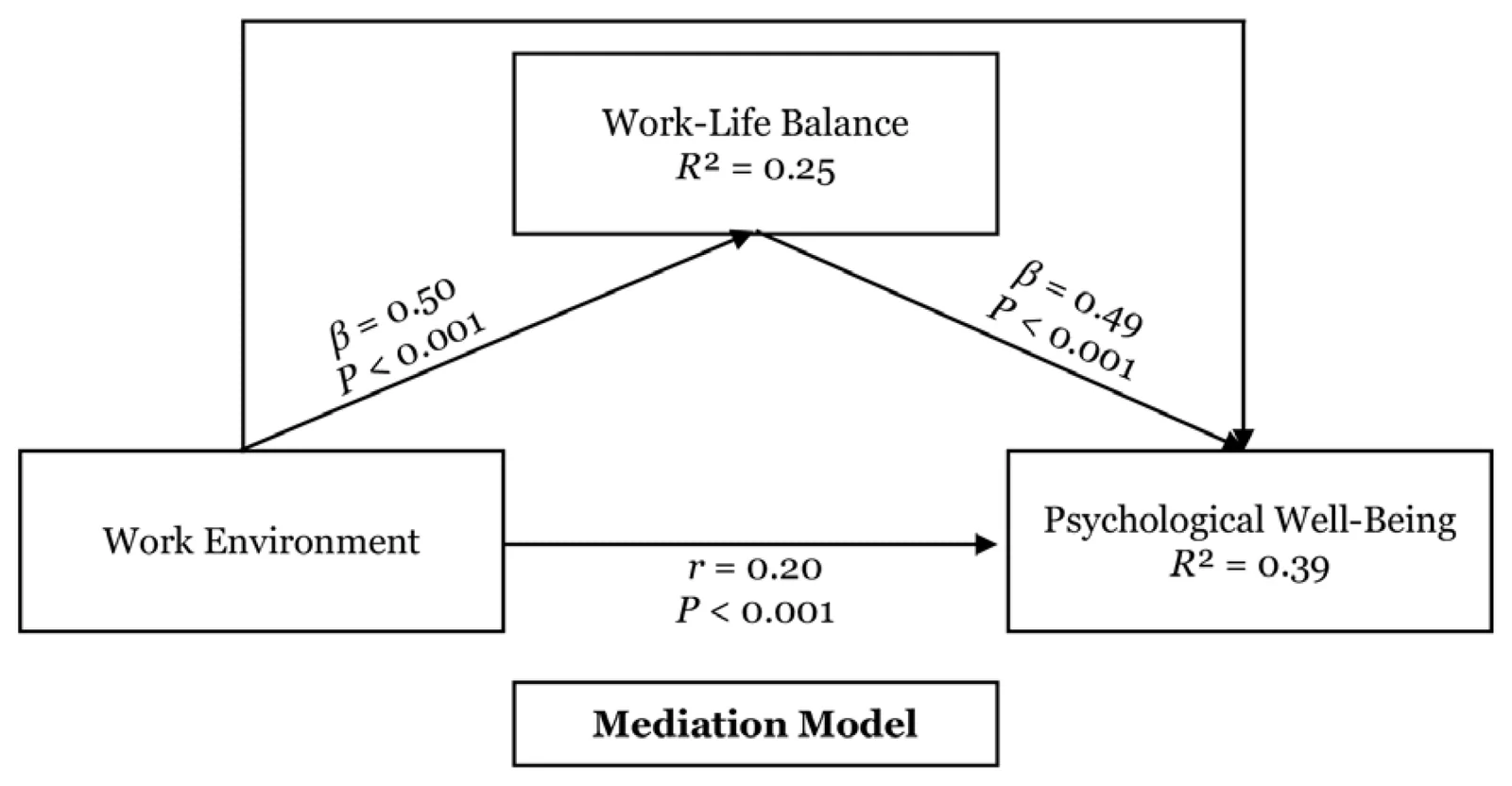
Mediation model of WLB on the relationship between work environment and PWB

**Table 1:** Characteristic of respondents

Variable	Category	Lecture *n* (%)	Admin staff *n* (%)	Total *n* (%)
Gender	Female	131 (57.2)	58 (49.2)	189 (54.5)
	Male	98 (42.8)	60(50.8)	158 (45.5)

Employment status	Civil servants	223 (97.4)	49 (41.5)	272 (78.4)
	Non-civil servants	6 (2.6)	69 (58.5)	75 (21.6)

Unit/Faculty	Teacher training and education	59 (25.8)	23 (19.5)	82 (23.6)
	Animal husbandry. marine and fisheries	20 (8.7)	5 (4.2)	25 (7.2)
	Social and political sciences	27 (11.8)	6 (5.1)	33 (9.5)
	Law	29 (12.7)	4 (3.4)	33 (9.5)
	Agriculture	21 (9.2)	16 (13.6)	37 (10.7)
	Science and engineering	21 (9.2)	6 (5.1)	28 (8.1)
	Public health	13 (5.7)	3 (2.5)	16 (4.6)
	Medicine and veterinary medicine	23 (10.0)	13 (11.0)	36 (10.4)
	Economics and business	16 (7.0)	2 (1.7)	18 (5.2)
	Postgraduate programme		3 (2.5)	3 (0.9)
	University bureaus		20 (16.9)	20 (5.8)
	Supporting institutions		10 (8.5)	10 (2.9)
	Technical service unit		7 (5.9)	7 (1.9)

**Table 2:** Percentile cut-offs for work environment, WLB and PWB

Variable	Very low	Low	Moderate	High
Work environment	< 105	105 to 117	117 to 129	> 129
WLB		< 54	54 to 67	> 67
PWB		< 82	82 to 95	> 95

**Table 3:** Average scores of the WESS-32 dimension

Dimension	Lecturers Mean (SD)	Staff Mean (SD)	Total
Autonomy	18.80 (3.60)	16.80 (3.60)	18.10 (3.70)
Stress and work pressure	16.10 (5.00)	16.20 (4.70)	16.10 (4.90)
Ethics	15.70 (3.50)	15.10 (3.00)	15.50 (3.40)
Managerial support	14.90 (4.10)	15.20 (3.20)	15.00 (3.90)
Role clarity	14.20 (3.40)	13.70 (2.90)	14.00 (3.30)
Social responsibility	10.90 (2.80)	10.90 (2.80)	10.90 (2.80)
Work practices	11.00 (2.20)	9.40 (2.30)	10.40 (2.40)
Commitment	8.00 (1.70)	7.70 (1.50)	7.90 (1.60)
Peer cohesiveness	7.60 (1.60)	7.60 (1.70)	7.60 (1.70)
WESS-32	112.30 (12.10)	108.00 (9.00)	110.80 (11.40)

WESS-32 = 32-item Work Environment Services scale

**Table 4:** Average scores of the WLB-15 dimension

Dimension	Lecturers Mean (SD)	Staff Mean (SD)	Total
Personal life enhancement of work	12.30 (2.30)	12.20 (2.40)	12.30 (2.40)
Work interference with personal life	11.00 (4.40)	11.30 (4.00)	11.20 (4.30)
Personal life interference with work	8.70 (3.50)	8.30 (3.50)	8.60 (3.50)
Work enhancement of personal life	7.40 (1.60)	7.00 (1.80)	7.30 (1.90)
WLB-15	59.90 (9.20)	59.30 (8.80)	59.70 (9.10)

WLB-15 = 15-item Work-Life Balance scale

**Table 5:** Average scores of the RPWBS-18 dimension

Dimension	Lecturers Mean (SD)	Staff Mean (SD)	Total
Self-acceptance	15.80 (2.10)	15.00 (2.60)	15.60 (2.30)
Personal growth	16.30 (1.80)	15.80 (2.10)	15.20 (2.60)
Positive relations with others	15.20 (2.40)	14.80 (2.40)	15.10 (2.40)
Purpose in life	13.90 (2.50)	13.10 (2.60)	14.60 (2.60)
Environmental mastery	14.50 (2.40)	13.90 (2.50)	14.30 (2.50)
Autonomy	14.00 (2.30)	13.00 (2.40)	13.70 (2.40)
RPWBS-18	89.90 (9.20)	85.60 (8.70)	88.40 (9.20)

RPWBS-18 = 18-item Ryff Psychological Well-Being Scale

**Table 6:** Mediation analysis

Measurement aspect	Category	Estimate	95% CI		*P*-value

			Lower	Upper	
Direct effect	WE – PWB	0.21	0.09	0.32	< 0.001
	WE – WLB	0.50	0.41	0.58	< 0.001
	WLB – PWB	0.49	0.39	0.59	< 0.001

Indirect effect	WE – WLB – PWB	0.25	0.19	0.31	< 0.001

Total effect	Direct Effect + Indirect Effect	0.46	0.35	0.55	< 0.001

Mediating proportion	WE – WLB – PWB	0.54			

Coefficient of determination (*R*^2^)	PWB	0.39			
	WLB	0.25			

Effect size (*f*^2^)	WE – PWB	0.05			
	WE – WLB	0.34			
	WLB – PWB	0.30			

Effect size (*v*)	WE – WLB – PWB	0.06			

CI = confidence interval; WE = work environment; WLB = work-life balance; PWB = psychological well-being
